# A new ultrafast energy funneling material harvests three times more diffusive solar energy for GaInP photovoltaics

**DOI:** 10.1073/pnas.2019198117

**Published:** 2020-12-14

**Authors:** Marcel M. Willich, Lucas Wegener, Johannes Vornweg, Manuel Hohgardt, Julia Nowak, Mario Wolter, Christoph R. Jacob, Peter Jomo Walla

**Affiliations:** ^a^Department for Biophysical Chemistry, Institute for Physical and Theoretical Chemistry, Technische Universität Braunschweig, 38106 Braunschweig, Germany;; ^b^Department for Theoretical Chemistry, Institute for Physical and Theoretical Chemistry, Technische Universität Braunschweig, 38106 Braunschweig, Germany

**Keywords:** ultrafast spectroscopy, pump–probe experiments, light-harvesting concentrators, polarization experiments

## Abstract

There is no theoretical limit in using molecular networks to harvest diffusive sun photons on large areas and funnel them onto much smaller areas of highly efficient but also precious energy-converting materials. The most effective concept reported so far is based on a pool of randomly oriented, light-harvesting donor molecules that funnel all excitation quanta by ultrafast energy transfer to individual light-redirecting acceptor molecules oriented parallel to the energy converters. However, the best practical light-harvesting system could only be discovered by empirical screening of molecules that either align or not within stretched polymers and the maximum absorption wavelength of the empirical system was far away from the solar maximum. No molecular property was known explaining why certain molecules would align very effectively whereas similar molecules did not. Here, we first explore what molecular properties are responsible for a molecule to be aligned. We found a parameter derived directly from the molecular structure with a high predictive power for the alignability. In addition, we found a set of ultrafast funneling molecules that harvest three times more energy in the solar’s spectrum peak for GaInP photovoltaics. A detailed study on the ultrafast dipole moment reorientation dynamics demonstrates that refocusing of the diffusive light is based on ∼15-ps initial dipole moment depolarization followed by ∼50-ps repolarization into desired directions. This provides a detailed understanding of the molecular depolarization/repolarization processes responsible for refocusing diffusively scattered photons without violating the second law of thermodynamics.

## Significance

Using molecular systems that harvest diffusive sunlight on large areas and funnel it onto much smaller areas of precious high-performance solar cells could pave the way to affordable high-efficiency photovoltaics. Here, we discovered important structural principles of molecules suitable to align diffusive light, the underlying ultrafast depolarization/repolarization dynamics, and a material with significantly higher light harvesting in the peak of the solar spectrum.

Silicon solar cells can never exceed efficiencies higher than about 30% as the electronic band gap of silicon is much lower than the energy of most solar photons (Shockley–Queisser limit) (1, 2). However, whereas multijunction solar cells with different band gaps can have much higher efficiencies (3–5), their material is exceedingly expensive. One solution could be using luminescent solar concentrators consisting of more affordable materials that collect light on large areas and concentrate it onto much less material of the high-efficiency energy converters. Simple lens or curved mirror solutions cannot serve as concentrators as a very large fraction of solar light is always diffusively scattered due to clouds or other objects. However, how can diffusively scattered photons be refocused without violating the second law of thermodynamics?

Nature provides evidence that this can be achieved by molecular networks of interconnected pigments that funnel their excitation energy by ultrafast energy steps to desired locations (6–11).

For photovoltaic applications, this concept can only work if the light-harvesting concentrators have efficiencies of nearly 100% as otherwise the higher efficiencies of expensive high-performance solar cells are quickly outweighed (12, 13). Therefore, researchers currently try to find systems that reach close to light-harvesting efficiencies of 100% (14–23). Conventional light-concentrator concepts consist of one-pigment composites in waveguiding materials such as poly(methyl methacrylate) and have several intrinsic loss mechanisms that quickly reduce their light-harvesting efficiency below 50%. Among the most important loss mechanisms of conventional concentrators are escape cone losses and reabsorption losses (24–28). Escape cone losses are due to preferential excitation of pigments with transition dipole moment orientations perpendicular to the waveguiding direction, resulting into reemission into directions not suitable for waveguiding. High reabsorption losses that occur during waveguiding to the photoconverter are also intrinsic to one-pigment light concentrators as the pigment concentration must be high enough to absorb the entire sunlight.

**Fig. 1.**
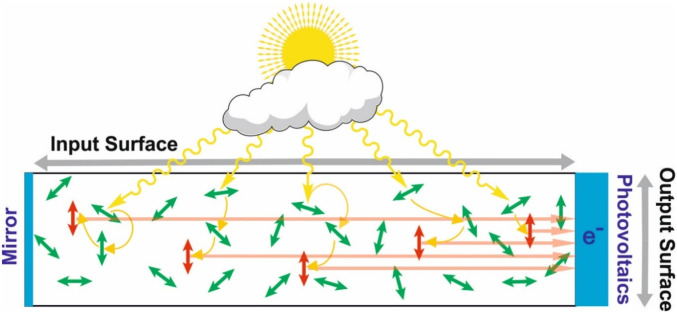
High-efficiency light harvesting and concentration. A pool of randomly oriented, light-harvesting donor molecules (green) absorbs sun light photons from all directions and funnels the excitation quanta by ultrafast energy transfer steps to individual light-redirecting acceptor molecules (red). The light-redirecting acceptor molecules are all oriented vertical and therefore emit almost all photons in directions suitable for total internal reflection wave guiding toward the energy-converting material at the output surface (29). The concentration factor is the ratio of the output surface divided by the input surface.

The best artificial system reported so far contains a pool of randomly oriented, light-harvesting donor molecules (green in Fig. 1) that funnel their entire excitation quanta by ultrafast energy transfer steps to individual light-redirecting acceptor molecules (red in Fig. 1) that are all oriented parallel to the energy-converting material (29). Experimental evidence was provided that this concept allows near to 100% quantum efficiency in harvesting photons from different directions and redirecting photon quanta in directions suitable for total internal reflection waveguiding and GaInP layers without significant reabsorption or escape cone losses. The potential cost reduction of such a combined light-harvesting/photovoltaic architecture depends very much on the concentration factor of the light-harvesting device, which is limited by the dimensions of the input surface and the output surface (Fig. 1, maximum concentration factor = input surface/output surface; note that in a cuboid the corresponding surfaces are proportional to the edge lengths as denoted in the figure).

The proof-of-principle material was discovered by empirical screening of pigments for whether they either were aligned or stayed randomly oriented during stretching of the exact same transparent polymers in which they were previously embedded. In the proof-of-principle system, a combined energy funneling and emission quantum efficiency of at least 85% was observed and 90% of these photons were redirected into directions suitable for total internal reflection waveguiding.

**Fig. 2.**
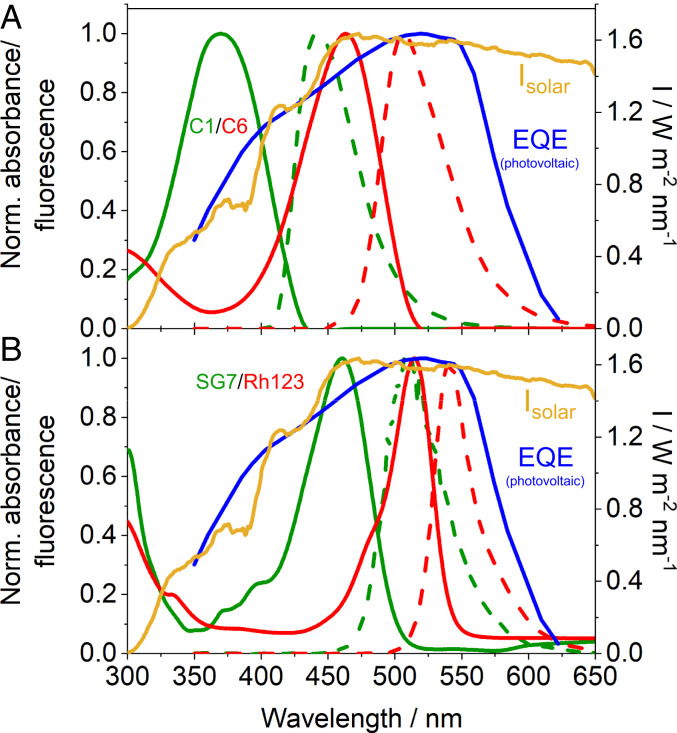
Spectral characteristics of donors, acceptors, the sunlight as well as photovoltaics. (*A*) Terrestrial solar energy irradiation spectrum (orange line) with absorption (solid green) and emission spectra (dashed green) of the light-harvesting donors as well as absorption (solid red) and emission spectra (dashed red) of the light-redirecting acceptors from previous light-harvesting systems [Coumarin 1/Coumarin 6, Pieper et al. (29)] along with the external quantum efficiency (EQE) spectrum of the blue GaInP layer of world record solar cells (blue line). While the light-redirecting acceptor emission matches very well with the EQE spectrum, the overlap of the light-harvesting donor spectrum with the solar spectrum is poor. (*B*) Corresponding spectra of the new Solvent Green 7/Rhodamine 123 (SG7/Rh123) light-harvesting system. Here, the absorption spectrum of the light-harvesting donor (green) has an excellent overlap with the maximum in the solar energy spectrum (orange), while the light-redirecting acceptor emission (dashed red) matches also perfectly with the EQE spectrum of GaInP photovoltaics (blue). EQE spectrum © 2016 IEEE. Reprinted with permission from ref. 5. Solar energy irradiation spectrum reprinted with permission from ref. 30, which is licensed under CC BY 4.0.

However, while almost all of the photons in the spectral absorption maximum of the donors were absorbed, its absorption maximum around 350–375 nm (green in Fig. 2*A*) contains three times less energy than in the maximum of solar energy irradiation spectrum at about 475 nm (Fig. 2*A*, orange line) (30). In other words, because the absorbing molecules in this system, Coumarine 6, absorb little in the peak of the solar spectrum, a corresponding larger thickness of the light-harvesting layer (directly affecting the size of the output surface in Fig. 1) is necessary to absorb all photons and therefore the concentration ratio of output surface/input surface is correspondingly lower. To compensate the low absorption by further increasing the concentration is not possible because of concentration quenching and other effects leading to losses. However, finding suitable light-harvesting molecules with higher extinction coefficients in the solar peak will directly increase the concentration factor as correspondingly thinner light-harvesting layers will suffice to absorb all photons.

When looking for molecular systems that better absorb solar light, the theoretical framework of anisotropic light absorption, energy transfer and light emission as well as fitting the correct wavelengths for the solar spectrum as well as all bandgaps of multijunction high-performance photovoltaic cell, including the lower bandgap components, is well known. However, the lack of knowledge about why a molecule aligns or not is very dissatisfying as this leaves only time-consuming empirical screening of many molecules. There was no obvious molecular property explaining why certain molecules would align very effectively whereas other very similar molecules did not. For example, Coumarine 6 aligned very well in stretched poly(vinyl alcohol), whereas the structurally related Coumarine 1 does not. Similarly, Rhodamine 123 (Rh123) aligned in the stretched polymer, whereas the structurally very similar Rhodamine 6G does not.

Therefore, we explored structural molecular parameters responsible for molecule alignment in stretched polymers as an important step for finding molecules with a better concentration factor for the blue GaInP layer as well as light-harvesting and light-redirecting molecules absorbing and emitting at longer wavelengths for the lower bandgap components of multijunction high-performance photovoltaic cells. A better understanding of this is also of interest for other potential applications of aligned molecules such as optoelectronics, optological circuits, communication technology, or emissive devices.

Here, we first report on insights into molecular structural elements that govern a molecules ability to be aligned in stretched polymers. We found that the alignability is the highest if the molecule contains linear band structures of rigid and planar rings. A higher number of structures pointing out of this planar band in any three-dimensional (3D) direction quickly lowers the alignability. This includes additional conjugated planar aromatic rings outside this band. A parameter observed from the ratio of atoms within the rigid linear band and atoms outside this band has a very high predictive power for the alignability, as experimentally confirmed by 27 molecules. These findings are practically further confirmed by a light-harvesting material that has a similar combined energy funneling, emission quantum redirectioning than the best previously reported system but that has a maximum light-harvesting absorption wavelength matching with the maximum of the terrestrial solar energy irradiation spectrum. It has therefore a three times higher possible concentration factor with an emission wavelength matching with the high conversion efficiency wavelengths of the GaInP layer of multijunction solar cells. Comparing femtosecond-energy transfer dynamics with donor excitation polarizations perpendicular and parallel to the acceptors allowed details insights into the ultrafast dynamics responsible for depolarization of excitation energy in the randomly oriented pool of light-harvesting molecules and subsequent repolarization by the aligned, light-redirecting molecules.

## Results

**Fig. 3.**
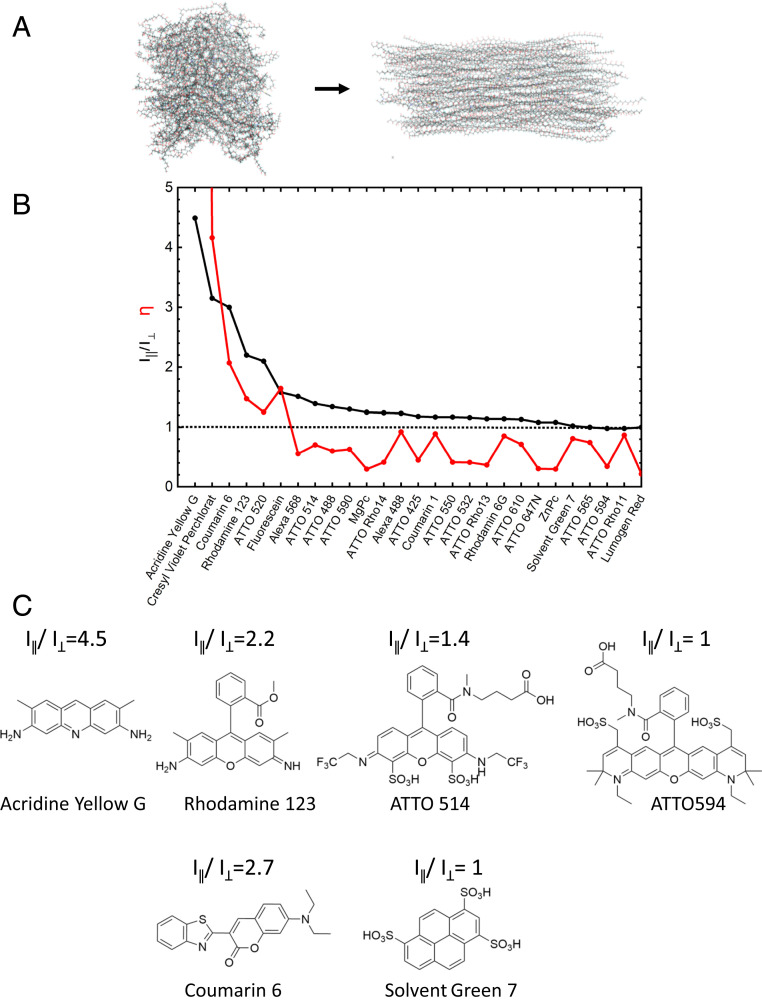
Alignability of different molecules in stretched polymers. (*A*) Visualization of a model system of polyvinyl alcohol polymer before and after stretching by a factor of ∼500%, based on molecular dynamics simulations. (*B*) Experimental alignabillty of the transition dipole moment of different molecules determined by fluorescence polarization observed parallel and perpendicular to the stretching direction, I_∥_/I_⊥_ (black, Eq. **1**) and structural predicting factor η determined from the numbers, N_Band_, and N_OutOf__Band_ as defined in the text (Eq. **2**). A value of η > 1 predicts molecular alignability. (*C*) Exemplary molecular structures and corresponding alignabilities I_∥_/I_⊥_ of six typical dyes.

First, we explored in more detail what molecular properties might be responsible for the ability to be aligned in stretched polymers. Various parameters were investigated, such as the presence and distance of polar groups, dipole moments, molecular mass or number of heteroatoms. Molecular dynamic simulations of stretched polymers provided a first indication that sterical effects could play a major role (Fig. 3*A*). In the following, we define the experimentally observed ability of a molecules transition dipole moment to be aligned in a stretched polymer by the fluorescence intensity emitted by the molecules with a polarization parallel to the polymer stretching I_∥_, divided by that with a polarization perpendicular to it, I_⊥_ (29):

$$\text{Alignability}=\;{\text{I}}_{∥}/{\text{I}}_{⊥}.$$ [1]

The higher I_∥_/I_⊥_, the larger the fraction of molecules with transition dipole moments in the direction of stretching. A value of I_∥_/I_⊥_ = 1 corresponds to unpolarized fluorescence emission after stretching, i.e., no alignment at all. A value I_∥_/I_⊥_ < 1, which we have observed only rarely so far, corresponds to a preferred transition dipole moment orientation rather perpendicular to the stretching direction.

Closer inspection of molecules that align very well provided indication that the highest ability of molecules to be aligned in stretched poly(vinyl alcohol) is connected to the length of linear bands of rigid ring structures (Fig. 3*C*). Apparently, the alignability is the highest, when the number of groups pointing out of this planar band is smallest. For example, Acridine Yellow G has the highest alignability that we found experimentally. The structure consists of three aromatic rings in a row and the methyl and amino groups can be regarded as rigid extensions within this band. Rh123 has also a high alignability, but the value I_∥_/I_⊥_ is already much lower than for Acridine Yellow G. ATTO 514 has already a value of I_∥_/I_⊥_ close to 1, even though it is structurally quite similar to Rh123. Another molecule with the same structural core, Atto 594, has basically a value of I_∥_/I_⊥_ of 1, even though it has a longer linear band of rigid rings. Obviously, a structure containing three aromatic rings in a row is very rigid and the forces occurring during the stretching very likely will align such molecules in the direction of stretching. If that same structure contains side groups pointing out the plane of the rigid band, the same forces seem to result also in other orientations during stretching. If the side groups are bigger, this effect is bigger, if they are smaller, still some tendency will exist that the forces orient the molecule in other directions than in the case of a pure linear rigid band of rings. We were surprised that other than intuitively expected planar π-systems such as the structure of Solvent Green 7 (SG7) did also not align very effectively in the stretched polymers. Apparently, the extended aromatic plane next to the longest linear row of aromatic rings results also in similar forces as the side groups in the above-mentioned examples leading to transition dipole moment orientations other than directly in the stretching direction. The molecule Coumarine 6, which aligns very well, needs some special attention. Albeit there is a single bond between the two parts of the molecule, crystal structures and molecular dynamics simulations show that they both stay near to coplanar, likely due to mesomeric effects, forming together a longer band of planar rings. Coumarine 1 contains also a linear band of rigid rings, but the length is much shorter than in Acridine Yellow G or Coumarine 6.

In summary, it seems that the alignability of a molecule is the better, the larger the longest band of rigid linear ring structures is within a plane in this molecule and the smaller the number of groups is that potentially point out of this band in any 3D direction. To test for this hypothesis, we defined a parameter based on this observation in the molecular structure. The parameter is defined as being the number of atoms found in the plane of the longest band of rigidly connected (aromatic) rings in a structure, N_Band_, divided by all atoms, N_OutOf__Band_, that are pointing out of this rigid band:

$$\text{η}=N_{\text{Band}}/N_{\text{OutOfBand}}.$$ [2]

**Fig. 4.**
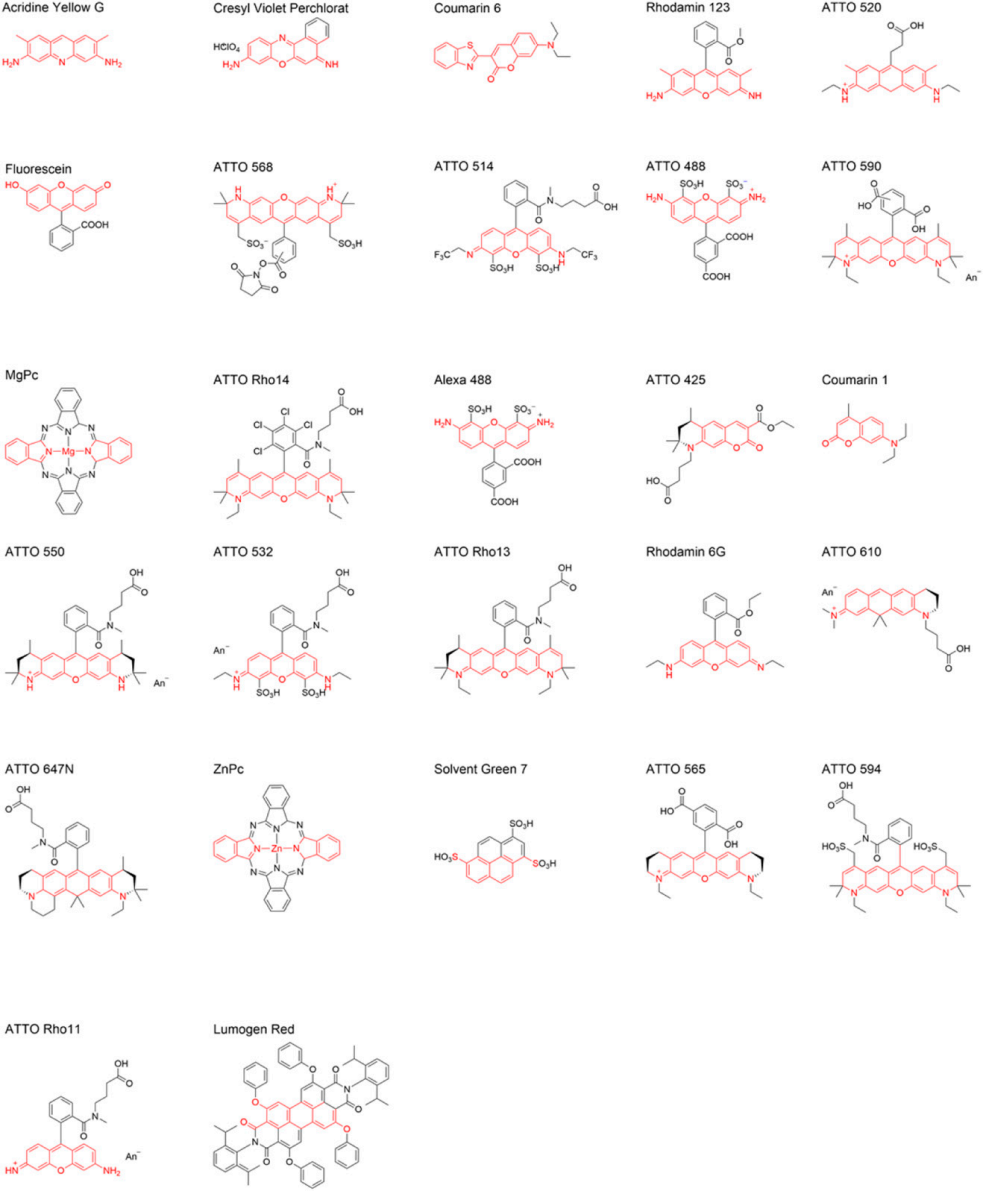
Molecular structures of the investigated molecules. (*Top Left*) Highest experimental alignability, I_∥_/I_⊥_. (*Bottom Right*) Lowest alignability, I_∥_/I_⊥_. The red and black colors denote distinct structural parts that were identified as promotors or inhibitors for molecular alignment, respectively (for details, see text). A more refined assignment for more complex molecules such as Lumogen Red will be subject of future studies.

Atoms belonging to incomplete rings at the ends of this band but which are still within the plane of the band and structurally similarly rigid as atoms within the band are also accounted to N_Band_ (see, e.g., Acridine Yellow G). Also, hydrogen atoms are counted with the atoms they are bound to. Fig. 4 illustrates the assignment of atoms to N_Band_ (red) and N_OutOf__Band_ (black) according to this definition for 27 molecules for which we also determined I_∥_/I_⊥_ experimentally. Fig. 3*B* shows a direct comparison of the parameter η with I_∥_/I_⊥_. This comparison demonstrates that η has a good predictive power for the ability of a molecular structure to be aligned during polymer stretching. If the number of atoms within the rigid band, N_Band_, outweighs the out of band atom number, N_OutOf__Band_ (η >1), then also significant experimental alignability is observed (I_∥_/I_⊥_ > 1.5).

This can help to find suitable molecules for the lower band gap layers as well as improved systems for the blue GaInP layer of current high-performance photovoltaics. Pigments that are aligned in the stretched polymer (η > 1, I_∥_/I_⊥_ > 2) are potential candidates for light-redirecting acceptors (red in Fig. 1), whereas pigments that preserve a random orientation in that same polymer during stretching (η < 1, I_∥_/I_⊥_ < 1.5) are potential candidates for light-harvesting donors (green in Fig. 1).

**Fig. 5.**
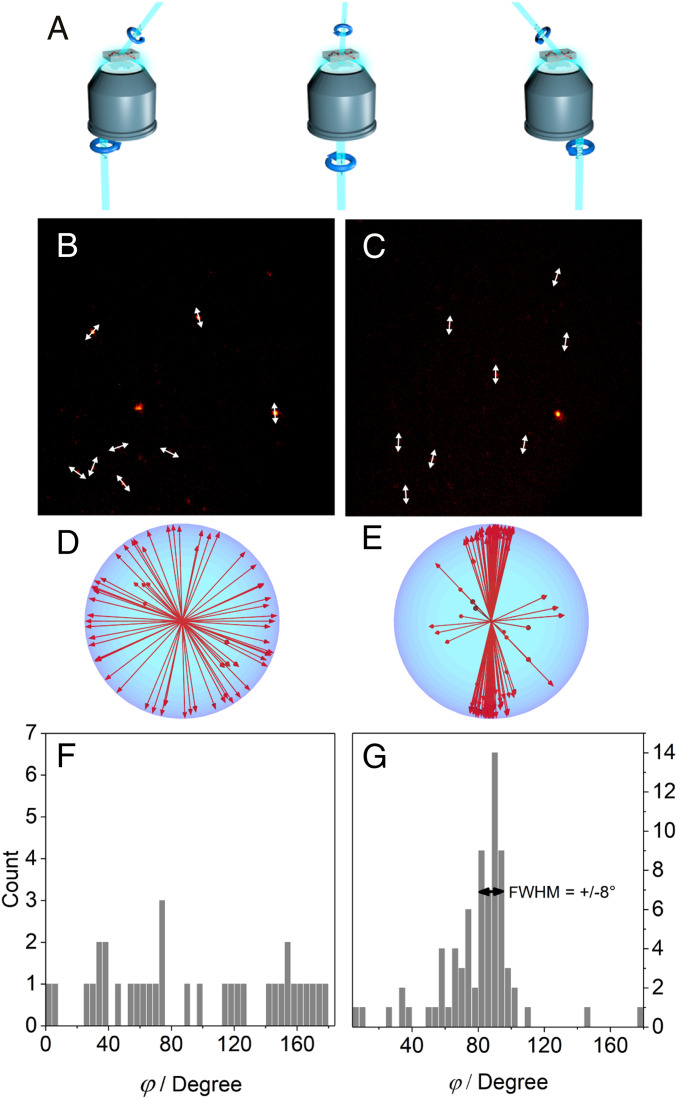
Three-dimensional single-molecule orientation measurements. (*A*) Rotating excitation polarization with tilted illumination from different directions allows to determine the 3D orientation of single molecules in a microscope setup. (*B* and *C*) Exemplary single-molecule images along with 2D transition dipole moment projections for acceptor molecules in a nonstretched and a stretched polymer, respectively. (*D* and *E*) Representation of the corresponding 3D orientations of a multitude of molecules. (*F* and *G*) Histograms of the individual angle deviations from the average.

The system presented here for GaInP layers was found based on these parameters and on the known spectroscopic properties of these molecules. The randomly oriented light-harvesting donor molecules SG7 in the system have values of η ∼0.8, I_∥_/I_⊥_ ∼1, whereas the light-redirecting acceptor molecules Rh123 have corresponding values of η ∼1.4, I_∥_/I_⊥_ ∼2.2 (Fig. 3*B*). The light-harvester absorption wavelength (green in Fig. 2*B*) matches perfectly with the maximum of solar irradiation energy (orange in Fig. 2*B*), the donor fluorescence (dashed green in Fig. 2*B*) has an ideal spectral overlap with the acceptor absorption (red in Fig. 2*B*) for highest energy transfer and funneling, and the acceptor emission (dashed red in Fig. 2*B*) matches ideally with the band gap of the photovoltaic material (overlap with external quantum efficiency [EQE] spectrum at the long wavelength edge, blue spectrum in Fig. 2*B*). The light-redirecting Rh123 has emission quantum efficiencies >90% (31).The 3D angular distribution of the dipole moment orientations of the light-redirecting acceptor Rh123 was confirmed using 3D-single molecule polarization microscopy (Fig. 5 and Movie S1) (29, 32). The full width at half-maximum in the corresponding angle histogram demonstrates an alignment precision of ±8° (Fig. 5 *C*, *E*, and *G*).

Next, the overall light-harvesting and energy redirection efficiency of the selected SG7/Rh123 donor acceptor system was optimized by varying the absolute concentrations of the light-harvesting donors SG7 as well the light-redirecting acceptors Rh123 as well as their relative ratio. Donor–acceptor ratios too low result in higher reabsorption losses due to high acceptor concentrations, whereas too high donor–acceptor ratios result in higher energy-transfer losses from light-harvesting donors further remote from the acceptors. Overall concentrations too low result in less than 100% light absorption (harvesting), whereas too high concentrations result in concentration quenching and reabsorption losses. Lower concentrations of the light harvesters can be compensated by larger light-harvesting optical path lengths but at the cost of the concentration ratio of the input and output surface (Fig. 1). Optimum initial donor–acceptor concentrations and ratios were computed with a custom-made ray-tracing software considering also molecular energy transfer and isotropic/anisotropic donor/acceptor absorption and emission (33). These values were then further optimized using angle-dependent photogoniometry with calibrated power meters detecting the fraction of the initially irradiated light energy redirected in the desired directions (29). We found a SG7 concentration of ∼2·10^−3^ M and Rh123 concentration of ∼2·10^−4^ M in the stretched polymer optimal, corresponding to a donor/acceptor ratio of 10 in the light-harvesting pools. These concentrations correspond to an average distance of the nearest donor, nD, to an acceptor of about 3 nm. This system had a combined donor energy funneling, transfer and acceptor emission quantum efficiency of ∼90% and redirects ∼90% of the photons in angle ranges suitable for total internal reflection waveguiding in materials of a refractive index of 1.5. The combined energy funneling, transfer and acceptor emission quantum efficiency was determined by the emission angle-dependent photogoniometry using calibrated power meters [see Pieper et al. (29) for details]. As the new material collects three times more energy per light-harvesting optical path length in the peak of the solar irradiance spectrum compared to previously published system [Pieper et al. (29)] with comparable efficiencies, it has a three times higher possible concentration factor (compare Fig. 2*A* with Fig. 2*B*).

**Fig. 6.**
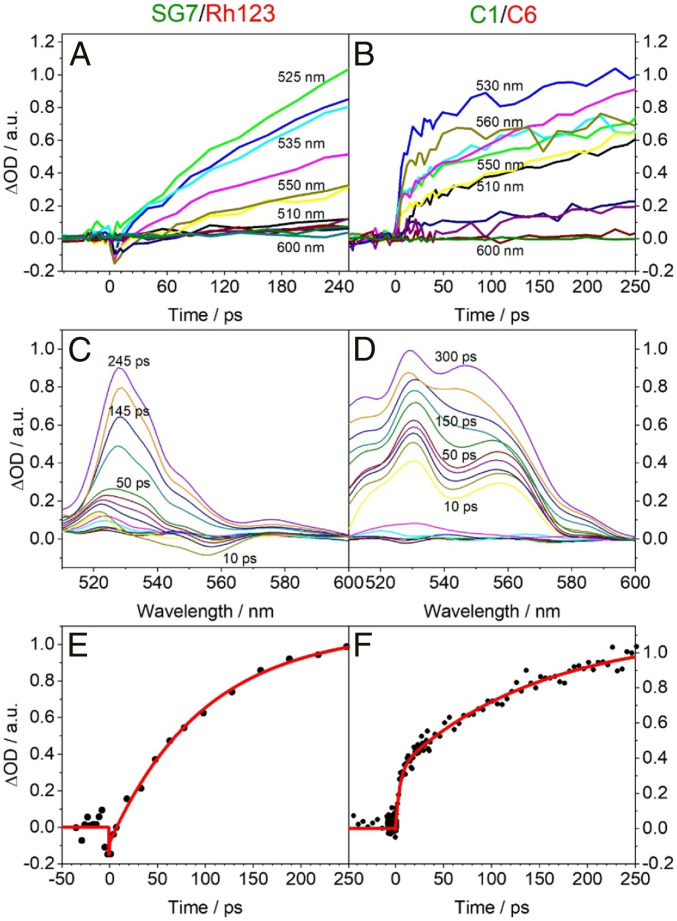
Pump–probe data of foils with pump polarization perpendicular to the probe polarization. (*A* and *B*) Time traces observed with various probe wavelengths for the unstretched SG7/Rh123 and C1/C6 system, respectively. (*C* and *D*) Corresponding transient absorption spectra at different time delays. (*E* and *F*) Corresponding time traces along with biexponential fits for the traces observed with a probe wavelength of λ_Probe_ = 530 nm. The pump wavelength was λ_Pump_ = 400 nm.

The high efficiency is based on the ultrafast energy-funneling and transfer as well as excited state dipole reorientation within the molecular network of the light-harvesting system (Fig. 1). Therefore, we explored the dynamics of the energy migration and dipole reorientation in the SG7/Rh123 as well the C1/C6 system in more detail by ultrafast spectroscopy. As expected, the migration of the energy to the light-redirecting acceptors can be observed on multiple timescales in the spectral region of acceptor absorption/emission after excitation in the spectral range of the light-harvesting donor absorption (Fig. 6). After about 200 ps, a transient signal builds up with a peak maximum around 530 nm in the Sg/Rh123 and 530 to 550 nm in the C1/C6 system (Fig. 6 *C* and *D*). As the entire light-harvesting system consists of a multitude of different donor/donor and donor/acceptor distances, we expect that this is reflected in a large range of different timescales in the kinetics. However, to characterize the approximate timescales, we performed a simple biexponential analysis and found characteristic timescales of around τ_nD→A_ ∼5 to 15 ps and τ_Dpool→A_ ≥100 to 200 ps (Fig. 6 *E* and *F*). The shorter timescale, τ_nD→A_, corresponds well with the timescale that one would expect from Försters theory for the transfer from the nearest donor molecules, nD, to the acceptors, whereas the longer timescale, τ_Dpool→A_, is likely due to donor pool energy migration and dipole moment reorientation. Please note that the exact timescales depend very sensitively on the average distances between the pigments in different preparations. While the overall light-redirecting quantum efficiency does not depend very much on minor variations in the concentration because the picosecond-energy transfer timescales are generally much shorter than the nanosecond-excited state lifetimes of the pigments, the energy transfer timescales themselves depend by 1/*r*^6^ on the interpigment distances. Thus, different preparations can easily display energy transfer timescales, τ_nD→A_, varying by a factor of 2 to 5 but since they are all well below the nanosecond-excited state lifetimes the energy transfer efficiency corresponds in all cases to near to 100%. Interestingly, we observed in general a significant decrease in the timescales in stretched polymers compared to the nonstretched systems. Even though the average distance between the pigments should not be changed due to stretching, we suspect that the pigments get closer in directions perpendicular to the stretching direction, which could open more energy transfer pathways that are faster and thus dominate the overall kinetics. In general, the overall kinetics of both systems are rather similar. The only qualitative difference in the SG7/RH123 system is the contribution of a fast kinetic with opposite sign in the transient spectra with a wavelength maximum around 555 nm (Fig. 6*C*).

**Fig. 7.**
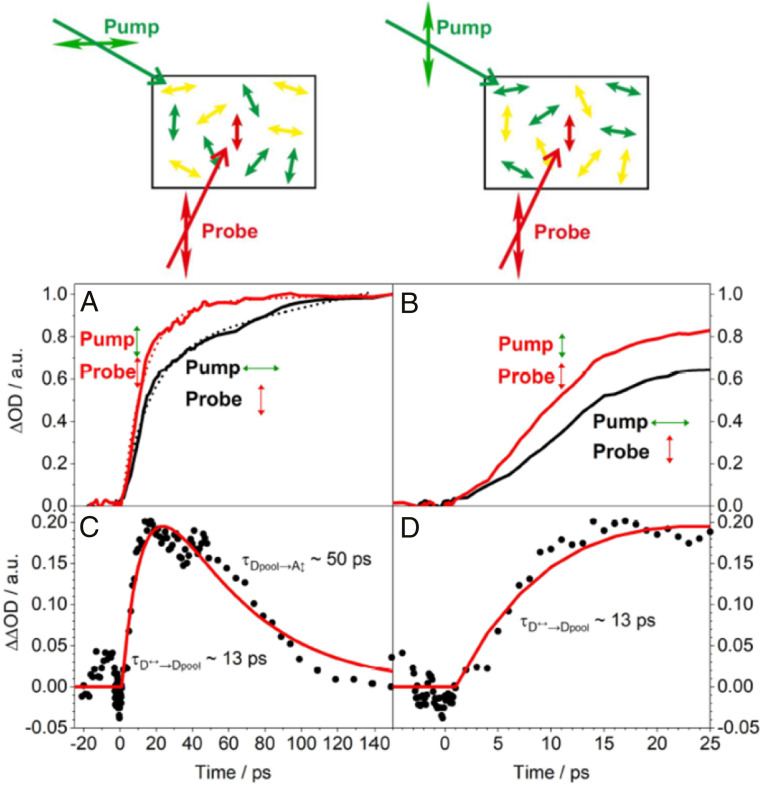
Polarized pump probe experiments with varying pump polarization. (*A* and *B*) Polarized pump–probe experiments of a stretched system with pump and probe polarizations parallel to the acceptor orientation (red solid line) and a corresponding trace with pump polarization perpendicular to the acceptor orientation and probe polarization (black solid line). The dotted red and black lines are corresponding biexponential fits in the range between 0 and 140 ps. Pump and probe wavelengths were λ_Pump_ = 400 nm and λ_Probe_ = 530 nm, respectively. (*C* and *D*) Kinetic traces calculated from the difference of the data observed in *A* and *B* with the pump beam oriented parallel and perpendicular to the acceptor alignment (black dots) along with a biexponential fit (red lines).

In order to separate the overall donor-pool energy funneling and donor-to-acceptor energy transfer dynamics from the dipole moment reorientation dynamics, we performed polarized pump–probe experiments with differing polarizations in the pump and probe beams (Fig. 7). As the SG7/Rh123 data contained contributions of the additional negative signals particularly at the important short timescales, we focused on the C1/C6 system that did not show these additional signals. When probing with a polarization parallel to the aligned acceptors, the signals observed after pumping the donors with a perpendicular polarization (Fig. 7*A*) increased significantly slower in comparison to the signals observed with parallel polarization (τ_nD↔→A↕_ > τ_nD↕→A↕_ ∼ 10 to 15 ps, black and red in Fig. 7 *A* and *B*). The longer timescales are larger than τ_Dpool↔→A↕_; τ_Dpool↕→A↕_ ∼ 200 ps. In Försters theory for intermolecular energy transfer, energy transfer is much more efficient when the dipole moments of donor and acceptor are parallel. The energy transfer efficiency becomes minimal the more the angle between donor and acceptor dipole moments come close to 90°. Therefore, the differences observed in Fig. 7 *A* and *B* are very likely due to the additional time that is necessary for donors with transition dipole moments perpendicular to the acceptors to first transfer their energy to other donors that have a transition dipole moment orientation in-between the dipole moments of the initial donor—and acceptors dipole moments.

The situation in the light-harvesting system typically corresponds to a preferred excitation of donors with a dipole moment perpendicular to the acceptor dipole moments because the sunlight from the top has a polarization that is rather horizontal (Fig. 1), whereas the polarization of the light perpendicularly redirected by the acceptors is rather vertical. Therefore, knowledge about this additional process—that is actually key to the high efficiency of the donor–acceptor systems presented in Fig. 1—is very important.

To further characterize the dynamics of this additional process, we calculated the difference between the kinetics observed with parallel and perpendicular donor excitation polarization (Fig. 7 *C* and *D*).

In a simplified manner, this kinetics corresponds to a consecutive kinetics in which the initially excited donors with a dipole moment perpendicular to the acceptors are the initial state (D↔), excited donors with a dipole moment in-between the dipole moments of the initial donor—and acceptors dipole moment are the intermediates (D_pool_) and the finally excited acceptor (A_↕_) is the final state. Therefore, we fitted a biexponential reaction kinetic scheme to the difference spectra (red lines in Fig. 7 *C* and *D*). We interpret the observed time constant for the rise term, τ_D↔__→__Dpool_ ∼13 ps, as the approximate timescale that is associated with the energy migration from initially excited donor molecules with preferential perpendicular dipole moments, D↔, to the entire randomly oriented light-harvesting donor pool, D_pool_. Correspondingly, we interpret the time constant for the decay term, τ_Dpool__→__A↕_, as the approximate timescale that is associated with the energy migration from the randomly oriented light-harvesting donor pool, D_pool_, to the light redirecting acceptor with a dipole moment parallel to, for example, a photovoltaic device, A_↕_. According to this interpretation, the energy on donors with perpendicular dipole moments needs about τ_D↔→Dpool_ ∼13 ps to migrate into the entire donor pool with isotropic dipole orientation and then about τ_Dpool→A↕_ ∼50 ps to be funneled to the light-redirecting acceptors with angular repolarized, highly anisotropic dipole moments perpendicular to the initially excited light-harvesting donor dipole moments.

## Conclusion

The comparison of molecular structure with the alignability of molecules seen experimentally in stretched polymers (measured by fluorescence polarization I_∥_/I_⊥_, Fig. 3 and Eq. **1**) provided very valuable insights into the question why the transition dipole moment of certain molecules aligns very effectively in stretched polymers, while in other, apparently very similar molecules it does not. A closer inspection indicated that the presence of longer, linear bands of rigid ring structures plays a major role and that pure planarity, such as in condensated polyaromates, is not the only decisive factor. Based on these observations, we propose a parameter, η (Eq. **2**), that is derived from the number of atoms within this planar band compared to the number of atoms outside this band. This parameter has a high predictive power for molecules that are potentially alignable in stretched polymers (Fig. 3*B*). For 27 molecules tested a value of >1 was a good indicator for molecules that aligned in stretched polymers (Fig. 3*B*). Certainly, the parameter can be refined in the future and different polymers might also influence the alignability in different ways, but a plausible relationship between alignability and molecular structure could be made. Of course, the alignability must be confirmed experimentally each time potential candidates are identified but the considerations presented here will facilitate the search for molecules that have this property. Molecular dynamics simulations to further validate our hypothesis and to identify possible additional indicators of the alignability are currently in progress.

The insights into the interconnection between structure and alignability of a molecule bridges the previous lack in knowledge when looking for ideal randomly oriented light-harvesting donor molecules and aligned light-redirecting acceptor molecules for the blue GaInP layer as well as lower band gap layers of current record photovoltaic cells. While the theoretical framework for the necessary isotropic/anisotropic light absorption and emission as well as intramolecular energy transfer processes was well known (33), very little was known about the necessary relation between structure and molecular alignment. Now, it was possible to find a combination of molecules suited much better for concentration of diffuse light in wavelength ranges for the spectral range of the blue layer (GaInP) of high efficient multijunction solar cells. While our previously published C1/C6 system had nearly 100% quantum efficiencies in energy transfer, light redirectioning and fluorescence emission matched quite well the band gap of the photovoltaic material (overlap with external quantum efficiency [EQE] spectrum at the long wavelength edge, blue spectrum in Fig. 2*A*), the light-harvesting donor molecules had a maximum absorption at ∼350–375 nm (green solid line in Fig. 2*A*), which is far from the maximum in the solar spectrum at ∼475 nm (orange in Fig. 2*A*).

A low absorption at 475 nm can be compensated by longer absorption path lengths but then the concentration factor intrinsically shrinks correspondingly, as the output surface becomes larger (output surface in Fig. 1) and consequently the gain in using less precious photovoltaic material is decreased. Note that a low absorption cannot be compensated by higher concentrations in an optimized system as this will directly increase reabsorption losses. Therefore, the only way to improve the concentration factor is to find light-harvesting molecules that have a significant higher absorption in the maximum of the solar spectrum at 475 nm. This is the case for the light-harvesting donor in the SG7/Rh123 system (green solid line in Fig. 2*B*), while the quantum efficiencies in energy transfer, light redirectioning, and fluorescence emission as well as match with the band gap of the photovoltaic material are at least as good as in the previous system. In the optimized system, it absorbs three times more light per optical path length at 475 nm, and therefore the potential concentration factor (output surface/input surface in Fig. 1) is correspondingly higher.

**Fig. 8.**
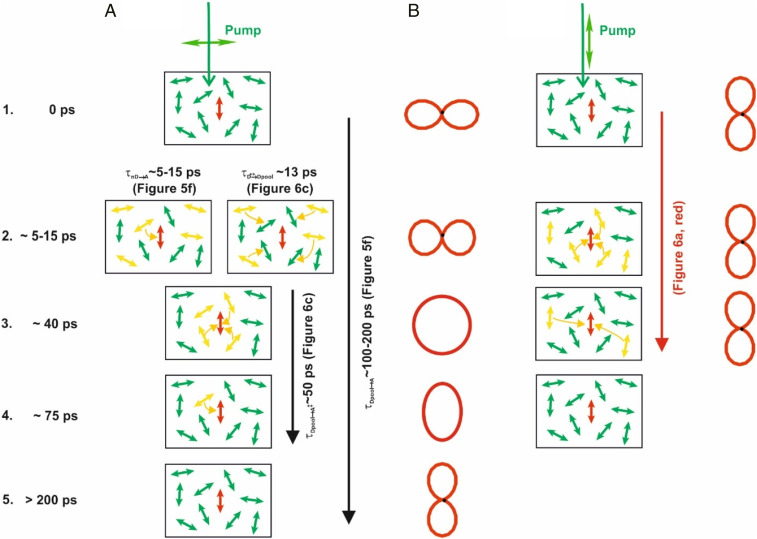
Scheme of the energy funneling and depolarization/repolarization mechanism, (*A*) Initial depolarization into the light-harvesting donor pool and subsequent repolarization into an orientation perpendicular to the initially excited transition dipole moment orientations. (*B*) Faster donor pool funneling observed with excitation polarization parallel to the acceptor transition dipole moments. For details, see text. The red lobes indicate the polarizations during steps 1 to 5.

Polarized pump–probe data (Figs. 6 and 7) allowed to dissect the mechanism (Fig. 8) for the ultrafast depolarizing light harvesting in the donor pool as well repolarizing funneling to the light-redirecting acceptors in detail.

When the polarization of the excitation light is perpendicular to the acceptors transition dipole moment (step 1 in Fig. 8*A*), light-harvesting donors are preferentially excited that have correspondingly horizontal transition dipole moments (step 2). This situation is close to the situation in light concentrators (Fig. 1). The preferential emission of these pigments into the same direction as the exciting light is an important reason for the high escape cone losses of conventional one-pigment light concentrators. In the present system, however, the excitation energy is transferred to the acceptors pigments with vertical orientations, first from the nearest donors pigments, nD (step 2). Simultaneously, more remote donors transfer energy to pigments of the donor pool, that have no preferred transition dipole moment orientation anymore (step 2). Both processes occur on a timescale of about 5 to 15 ps (τ_nD→A_ ∼5 to 15 ps and τ_D↔→Dpool_ ∼13 ps, respectively). The remaining excited donor pigments have now random orientations. In contrast to the initially preferential excitation of pigments with horizontal transition dipole moments, the polarization is lost (step 3). Next, this excited donor pool transfers also its energy to the aligned acceptors on a timescale of about τ_Dpool→A↕_ ∼50 ps. Depending on the relative orientation with the acceptor, donors with rather parallel transition dipole moment orientations transfer faster than others, that span rather larger angles (step 4). Finally, all donors have transferred their energy to the acceptors (step 5). The polarization is highly anisotropic again, but now it is exactly perpendicular to the initial excitation polarization. Consequently, the photons are now emitted perpendicular to the initial excitation on timescales larger than about 200 ps.

When using a donor excitation polarization already parallel to the acceptors (Fig. 8*B*, step 1), the kinetics are more simple and faster because there is no time necessary for donors with horizontal transition dipole moments to first transfer their energy to other donors that have transition dipole moment angles closer to the acceptors dipole moments. The donors that are already excited nearer to the acceptors are also more likely to have a preferential dipole moment orientation parallel to the acceptors (step 2). Thus, a larger contribution of faster timescales is seen that corresponds to what one would expect from Försters theory for the transfer from nearest donor molecules to the acceptors with rather parallel transition dipole moments (red curve in Fig. 7*A*). Also, more remote donors have already preferential dipole moment orientations that are rather parallel to the acceptor dipole moment orientation, and thus the energy transfer occurs also faster from this donor pool (step 3). The data in Fig. 7*A* (red curves) illustrate that the energy transfer is almost complete on timescales (∼40 ps) at which still reorienting energy funneling takes place when exciting with horizontal polarization (Fig. 7*A*, black curve, and Fig. 6).

The insights on the relationship between structure and alignability of a molecule (Figs. 3 and 4) and de/repolarization of light (Figs. 7 and 8) will help to find not only molecules, systems, and ultrafast light-steering and repolarization molecular networks to redirect and concentrate the full solar spectrum onto each layer of high efficiency solar cells but will also be useful for other systems in which aligned molecules can be very advantageous, for example, in optoelectronics, optical logical circuits, communication technology, emissive devices, as well as up- and down-conversion devices.

## Materials and Methods

Similar setups for 3D single-molecule microscopy, pump–probe spectroscopy, as well as photogoniometry have been described previously (29). More details as well as details of the preparation of the samples as well as polarized pump–probe experiments that were not already described in *Results* can be found in the extended supporting methods in *SI Appendix*.

## Supplementary Material

Supplementary File

Supplementary File

## Data Availability

All study data are included in the article and supporting information.
