# Supplementary figures and images for: A new ultrafast energy funneling material harvests three times more diffusive solar energy for GaInP photovoltaics
Source: Proc Natl Acad Sci U S A. 2020 Dec 14;117(52):32929–38. doi: 10.1073/pnas.2019198117 (PMC7776598; doi:10.1073/pnas.2019198117)

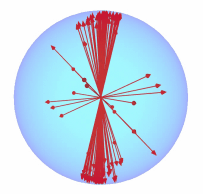

Supplement: Supplementary File [file pnas.2019198117.sm01.gif]
