# Supplementary material for: A new ultrafast energy funneling material harvests three times more diffusive solar energy for GaInP photovoltaics
Source: Proc Natl Acad Sci U S A. 2020 Dec 14;117(52):32929–38. doi: 10.1073/pnas.2019198117 (PMC7776598; doi:10.1073/pnas.2019198117)
Supplement: Supplementary File [file pnas.2019198117.sapp.pdf]

Supporting Information

Sample preparation:

First, a dye solution was obtained by dissolving 0.0210 g (2×10^{-3} M) of Solvent Green 7 and 0.0015 g (2×10^{-4} M) of Rhodamine 123 in 20 mL of dimethyl sulfoxide (DMSO). Then 2.2 g PVA (vh, lw) were added to the heated solution. The resulting solution was heated under stirring at 70 °C for 3h in N_2 atmosphere. Approximately 4 g of the mixtures were put on a glass plate and placed in a drying cabinet for 3 days at 50 °C under 200 mbar. By stretching the dye-doped foil by 400 % an orientation of the acceptors was received while the donors kept random orientation. The resulting foils showed a thickness of around 50-60 µm.

Determination of alignability, $I_{\parallel}/ I_{\perp}$, via polarized fluorescence spectroscopy

The determination of $I_{\parallel}/ I_{\perp}$ (eq. 1) has been described previously (30). Supplementary Figure 1 exemplary demonstrates the schematic fluorescence for the light-harvesting pigments and the light-redirecting pigments of the Coumarin 1 / Coumarin 6 system (C1/C6 in the following). The fluorescence intensities with polarizations parallel, $I_{\parallel}$, or perpendicular, $I_{\perp}$, to the stretching direction changes only very little for the light harvesting donor (SI Appendix, Fig. S1 a) whereas the corresponding fluorescence intensities of the light harvesting acceptors (SI Appendix, Fig. S1 b) differ significantly after stretching. For details see (30).

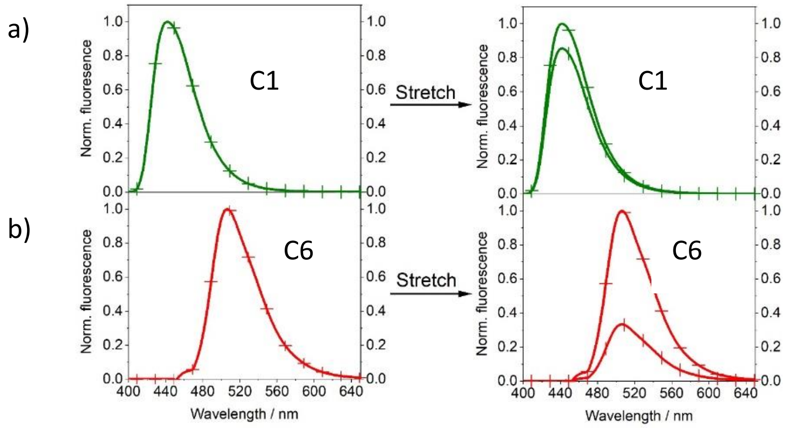

Fig. S1. Schematic and exemplary representation for determining alignability, $I_{\parallel}/ I_{\perp}$, via polarized fluorescence spectroscopy, for the C1/C6 system

a) While the parallel, $I_{\parallel}$,, and perpendicular components, $I_{\perp}$, of the light harvesting donor emission does not change significantly, the emission of the light-redirecting acceptors (b) is significantly larger with polarisations parallel to the stretching direction than perpendicular to it. Exemplary data for the for the C1/C6 system. The fluorescence data with horizontal bars (–) represent the $I_{\parallel}$ spectra whereas the data with the vertical bars (|) represent the $I_{\perp}$ spectra.

Three-dimensional single-molecule orientation measurements:

For the three-dimensional single-molecule measurements an acceptor only foil with a concentration of $5x10^{-11}$ M was prepared as described above. The single-molecule set up has been described previously.(1,2) However, the beam was focused on three different positions of the back aperture instead of 2 different positions. This enabled axial angles to be resolved even better. In addition, the λ/2 was used in a motorized rotation stage (STANDA) for the continuous rotation of the polarization vector.

Angle-dependent absolute power measurements:

A 485 nm pico-second pulsed diode laser (LDH-P-C 485 from PICO-QUANT) was used as a light source to excite the donor molecules. A short pass excitation filter (FES500 from THORLABS) was used, while in front of the detector an acceptor band pass emission filter (ET band pass 535/50, AHF) was placed. The further set up has been described previously.(1)

Pump–probe measurements:

A high repetitive Laser system (Coherent OPA/RegA operated at 120 kHz) was used for the pump-probe experiments, as described previously in (1). To increase overall stability, the RegA-pump-laser was replaced by a Verdi G Series (components from COHERENT INC.). A $\frac{\lambda}{2}$-waveplate (achromatic $\frac{\lambda}{2}$-waveplate, 400–800 nm, THORLABS) was used in both beampaths in front of the sample holder for the polarization measurements. Two band pass emission filters (ET band pass 535/50, AHF and ET band pass 535/30, AHF) were used instead of the Czerny-Turner Spectrometer to improve the signal to noise ratio in the spectroscopic measurements.

Movie S1. 3-Dimensional representation of the transition dipole moment orientations of Rhodamine 123 molecules aligned in a streched polymer.

References

1. Pieper A *et al. (*2018) Biomimetic light-harvesting funnels for re-directioning of diffuse light. *Nat. Commun.* 9:666.
2. Pfennig D, Albrecht A, Nowak J, Walla PJ (2020) A device for exploring the full angular excitation space – Can more angular projections improve determination of a molecules 3D-orientation in the presence of noise? *Chem. Phys.* 538:110853.
